# Sports nutrition knowledge and perceptions among professional basketball athletes and coaches in Lebanon-a cross-sectional study

**DOI:** 10.1186/s13102-021-00280-6

**Published:** 2021-05-17

**Authors:** Jocelyne Matar Boumosleh, Catherine el Hage, Antoine Farhat

**Affiliations:** Faculty of Nursing and Health Sciences, Notre Dame University-Louaize, Zouk Mosbeh, Lebanon

**Keywords:** Basketball, Nutrition, Knowledge, Coaches, Players, Lebanon

## Abstract

**Background:**

Basketball is the most popular sport in Lebanon. Adequate nutrition has been established to be a key component of optimal athletic performance, recovery from exercise and exercise-induced injury and documented to be associated with adequate nutrition knowledge (NK). In Lebanon, nutrition education is not incorporated into the basketball player training program and there is no established position for sports nutritionists in basketball clubs. To our knowledge, the present study is the first to evaluate the NK status of Division I Basketball (D1B) players /coaches in Lebanon. The objectives of this study are to assess the prevalence of inadequate NK; identify the gaps in NK, main sources of nutrition information, perceptions on sports nutrition and independent predictors of inadequate NK among D1B players and coaches in Lebanon.

**Methods:**

All D1B players (*n* = 184) and coaches (*n* = 16) in Lebanon were invited to participate in the study. Study participants were asked to complete a questionnaire that included questions on NK, resources and perceptions. A percentage of ≥60% of NK questions answered correctly was used as indicative of having adequate NK. Descriptive statistics were used to summarize the sample characteristics. The T-test and chi square test were used for comparisons of means and proportions, respectively. Logistic regression was used to explore the predictors of inadequate NK in D1B players.

**Results:**

The sample consisted of 178 D1B players (n_M_ = 126; n_F_ = 52) and 11 male coaches, resulting in survey response rates of 97 and 69%, respectively. Inadequate NK was found among about 80 and 54% of D1B players and coaches, respectively. Inadequate NK was found to be independently associated with lack of nutrition education in D1B players.

**Conclusions:**

Despite widespread lack of adequate NK among D1B players and coaches in Lebanon, our sports clubs do not have dietitians. Basketball sports clubs in Lebanon should start to budget for hiring a dietitian or carrying out nutrition education campaigns that are based on analyses of incorrect responses of our study participants. Findings of this study are of tremendous significance to D1B players in Lebanon in terms of improving the athletes’ physical health and performance.

**Supplementary Information:**

The online version contains supplementary material available at 10.1186/s13102-021-00280-6.

## Background

Basketball is a fast-paced group activity, represented by irregular episodes of high-force movement repeated over a prolonged time, and players utilize both aerobic and anaerobic energy systems. As a competitive team sport, basketball requires vigorous preparation for the very high-intensity rivalry games. A professional basketball player trains 2 to 3 h a day, 4 to 6 times each week, making basketball a high calorie-burning sport with significant physiological demands.

The Lebanese Basketball federation (FLB) organizes the top-tier professional men’s and women’s basketball teams in Lebanon. The Lebanon men’s and women’s national basketball teams have been considered among the top teams in Asia. National championships are organized annually by the FLB, with playoffs played and the winning team receiving a national cup. Players have a long pre-season and a normal season composed of 25 to 30 games in addition to tournament games. The two teams that advance play a best of four out of seven games in the final to determine the league champion. The season starts in December and ends in May.

The American Dietetic Association, Dietitians of Canada, and the American College of Sports Medicine recommend appropriate selection of food, fluids, and supplement choices as well as optimization of the composition and timing of intakes of macronutrients, micronutrients, and fluid throughout the day by athletes for provision of sufficient energy, replenishment of glycogen stores, maintenance of circulating blood glucose levels, build, maintenance and repair of tissue and ultimately enhancement of athletic performance, recovery from exercise and exercise-induced injury [1]. These recommendations are based on the best evidence available integrated with expert judgment. Recent studies found that a planned scientific dietary approach helped non-elite runners and endurance-trained cyclists complete a marathon run and a time trial faster, respectively [2, 3]. Studies showed that carbohydrate (CHO) loading not later than 4 h prior to, CHO consumption between 30 and 80 g/h during and within 2 h after endurance exercise significantly delay onset of fatigue and improve endurance performance [4–7]. This is attributed to the fact that inappropriate carbohydrate intake or loading prior to endurance exercise causes depletion of glycogen, the body’s predominant source of energy during moderate to high-intensity exercise. In a study that aimed to influence dietary intake and body composition to improve athletic performance of elite and subelite male Australian football and soccer players, adequate dietary intake was indicated to be positively correlated with fat-free soft tissue mass [8]. In a study done on nine trained cyclists to compare the effects of a carbohydrate and a carbohydrate-protein supplement on aerobic endurance performance, researchers found that the addition of protein to a carbohydrate supplement enhanced aerobic endurance performance above that which occurred with carbohydrate alone [9]. In a cross-sectional study among 293 Taekwondo players of Kathmandu Metropolitan City, it was shown that fat and energy intake have significant positive correlations with handgrip strength of athletes and energy intake has an independent positive association with athletic performance (β = 0.13, 95% CI: 0.12 = 0.14) [10]. Substantial evidence also exists that micronutrients play an important role in several physiologic processes, including energy production, oxygen transport, bone health, immune function, antioxidant function, fluid balance and build and repair of muscle tissue following exercise, that are crucial for peak endurance exercise performance. Several studies showed that marginal micronutrient deficiency states have potential implications for the overall health of athletes and performance. For example, iron-depleted endurance athletes have shown an impairment in exercise performance in several studies [11, 12]. Calcium losses, produced by a drop in levels of calcium-regulating hormones that results from intense endurance training with insufficient calories to meet training demands, predispose an athlete to stress fractures, osteoporosis and inevitably interfering with sport performance [13]. Female endurance athletes with low energy availability, menstrual dysfunction and low calcium intake are at greatest risk of developing the female athlete triad (disordered eating, amenorrhea, osteoporosis) [14] Similar to the adverse effects of macro- and micro- nutrient deficiencies on athletic performance, dehydration has been associated with increased risk of oxidative stress and compromised exercise performance [15]. Studies showed that fluid losses of 2% or more of body mass is sufficient to cause a noticeable decrease in performance, especially in warm-hot weather [16]. Maintaining proper hydration by drinking beverages containing carbohydrate and electrolytes during and after training or competition by endurance athletes, on the other hand, has been shown to delay fatigue, enhance performance and reduce risks of injury associated with dehydration and sweat loss [17].

Despite the well reported importance of a balanced diet for the athlete’s overall health, athletic performance, recovery from exercise and exercise-induced energy, research suggests that dietary Intake of athletes do not meet sport nutrition recommendations. The bulk of the literature explains this phenomenon by the lack of nutrition knowledge (NK) among athletes and its association with unhealthy dietary habits. In a cross sectional study surveying 73 Brazilian adolescent professional soccer players aged 14–19 years, the study participants showed a low NK (54.6%) and an inadequate intake of fruits, vegetables, dairy, carbohydrates, and micronutrients. In addition, sodium intake was negatively correlated with the three categories of the NK test (Basic NK (BNK), Sports NK (SNK), and Food Pyramid NK (FPNK), and phosphorus and calcium intakes were positively correlated with FPNK and both SNK and Total NK, respectively [18]. In another cross-sectional study done on 293 Taekwondo (TKD) players of Kathmandu Metropolitan City, it was found that more than half (56.3 and 55.6%) had poor NK and nutrition practice scores, respectively. The median energy (2368 Kcal), protein (79.5 g), fat (71.2 g), calcium (416 mg), and iron (7 mg) intake among TKD players were significantly lower than the corresponding Recommended Dietary Allowances for athletes [10]. In a study assessing the dietary intake, NK and hydration status of Irish Gaelic footballers (*n* = 168), findings suggested that the participants lack NK (40.2 ± 12.4%, *n* = 24) and had sub-optimal dietary practices. Dietary assessment showed an energy deficit (485 kcal [IQR 751, 6]) (*p* < 0.001), with carbohydrate intake (3.6 g/kg [IQR 3.0,4.1]) below current guidelines for athletes participating in 1 h moderate intensity exercise per day (5–7 g/kg; *p* < 0.001). Average vitamin D (3.8 μg [IQR 1.8, 5.5]) and selenium intakes (54.2 μg [47.2, 76.7]) were significantly below the reference nutrient intakes. In addition, a high proportion of athletes also had sub-optimal vitamin A (38.7%), potassium (30.6%), zinc (25.8%), magnesium (19.4%) and calcium (12.9%). Pre-exercise hydration status (median urine specific gravity 1.010 [IQR 1.005, 1.017]) was significantly below the cut-off to represent dehydration (1.020) [19]. In five studies done on Australian athletes to evaluate and compare nutritional intake to current recommendations, the study participants were found to have low NK (answered only 51, 57, 54.5%, 46, 56.9% (professional athletes) /61.3% (semiprofessional athletes) of the Sports NK Questionnaire questions correctly, respectively) and their energy and nutritional intakes of macronutrients (carbohydrate and protein) and some micronutrients (calcium and iron) were incoherent with current recommendations [8, 20–23]. The associations between poor NK and inadequate energy and nutritional intakes were demonstrated by the moderate positive correlations found between NK scores and meeting estimated energy requirements (r = 0.33, *P* = 0.03), protein (r = 0.35, *P* = 0.02), fiber (r = 0.51, *P* = 0.001, calcium (r = 0.43, *P* = 0.004 [22] and carbohydrate intakes (*n* = 41, ρ = 0.32, *p* = .04) [23]. In a cross-sectional study conducted on a group of 1040 British adults to investigate the relationship between NK and intake of fat, fruit and vegetables, researchers found NK to be an independent predictor of healthy eating habits; specifically, study participants in the highest quintile for NK were found to be almost 25 times more likely to meet current recommendations for fruit, vegetable and fat intake than those in the lowest quintile, after controlling for demographic variables [24]. Similarly, another study involving 21 professional rugby league players found overall NK to be positively correlated to consumption of fruit and vegetables (rs = .52, *p* < .05). In addition, a significantly higher percentage of rugby players in the good nutritional knowledge group reported consuming starchy foods, fruit & vegetables, oily fish and milk more frequently whereas, a higher percentage of players in the poor nutritional knowledge group reported more frequent consumption of fizzy drinks and squash [25]. A cross-sectional study done to evaluate the NK and dietary practices of postmenopausal women showed moderate positive significant correlations between NK and milk score, servings of high-calcium foods and food frequency score and a negative significant correlation between NK and the use of carbonated beverages [26]. Another study showed positive significant correlations of NK with intake of cereals (*r =* 0.30) and fruit and vegetables (*r =* 0.33) and negative significant correlations of NK with intake of fats and oils (*r* = − 0.38), tea and coffee (=*r*-0.31) and junk foods (*r =* − 0.29) [27]. A study done to assess dietary habits and practices of 383 NCAA Division I athletes, found that those who reported working with a sports dietitian for the purpose of dietary planning had a better understanding of the need to periodize nutrient intake by adjusting caloric intake according to demands of the training cycle (47.12% vs. 32.85%), were more likely to have school-provided boxed meals while on team trips (21.29% vs. 6.77%), and also less likely to consume fast food prior to practice or competition while on team trips (9.90% vs. 19.55%) [28]. Research also supported an association between inadequate NK and risk of eating disorders. In a longitudinal study done to assess NK, attitudes and eating behavior of 22 runners, it was found that runners with acceptable eating attitude test (EAT) scores (at low risk of eating disorders) had a mean of 70% on the NK test and consumed an average of 1781 kcal, while those with high EAT scores (at high risk of eating disorders) had a mean of 57% on the NK test and consumed an average of 1295 kcal daily [29]. In addition, multiple randomized clinical trials showed that nutrition education has been shown to be effective in improving dietary habits of athletes. In a recent experimental study that aimed to determine the effects of sports nutrition education intervention program on improvements in sports NK, attitude and practice, and dietary intake among Malaysian team sports athletes, significant increases in the intervention group’s (IG’s) mean scores for knowledge, attitude and practice compared to decreases in the respective mean scores of the control group were observed. In addition, significant improvements were found in the IG’s total energy intake, total carbohydrate and total protein intake compared to those of the control group [30]. In another trial done to evaluate the effectiveness of nutritional counselling in improving the athlete’s NK, body composition and eating behavior, it was found that the intervention resulted in increased NK, lean body mass, intake of vegetables and fruits and decreased intake of sweets and oils [31] . In another experimental study done to assess the effectiveness of nutrition education in active middle school and high school students, significant improvements on questions about protein and exercise, and dietary supplement use knowledge were observed in both middle and high school students. In addition, knowledge on general nutrition, fiber, hydration, vitamins and minerals improved significantly in high school students only, whereas, knowledge on carbohydrates improved significantly in middle school students only [32] . An intervention study investigating the effect of a six-month nutrition education program on eating behavior and skeletal loading exercise in competitive male road cyclists at risk for relative energy deficiency in sports, found those with increased energy availability over the 6-month intervention period to have improved bone health and race performance than those with reduced energy availability [33].

Acquiring adequate and credible nutrition information is important as it would shape the athlete’s NK and, therefore, could inform nutritional choices which, in turn, would mold the athlete’s physical health, athletic performance, recovery from exercise and exercise-induced injury. Several studies showed that the nutrition information sources are not equally accurate and therefore do not all have a positive impact on NK. In cross-sectional studies done to examine the relationship between sources of nutrition information and NK, it was found that use of healthcare professionals, nutritionists/dietitians, books and/or magazines, or newspapers as a source of nutrition information was positively associated with NK [34, 35], whereas, use of physicians/nurses and television/radio as sources of nutrition information had a negative impact on NK [36]. The unavailability of sports nutritionists in the athletes’ clubs makes athletes often turn to readily accessible sources of sports nutrition information such as the coaches, assistant coaches (ACs), athletic trainers (ATs), strength and conditioning coaches (SCCs), internet, parents/friends and social media. In a cross-sectional study done to assess the NK status of 100 athletes enrolled in a NCAA Division II sport, athletes reported school (25%), online sources (24%), parents/friends (19%), personal experience (10%), personal research (10%), coaches (8%) or ATs (4%) as their primary source of nutrition information [36]. In another survey involving 185 athletes enrolled in NCAA Division I, II, and III institutions, athletes indicated their top three choices of nutrition information as SCC (16.2%), AT (11.4%) and coach (7.7%) [37]. In a cross-sectional study that surveyed 207 Iranian basketball and football college players, the players reported coach as the primary nutrition information source (89.4%), television, radio or the Internet (59.9%) as the second most and a family member or friend (27.5%) as the third most popular source of nutrition information [38] . In a study involving NCAA division I, II or III collegiate cross-country runners, runners reported that their top four sources of nutrition information were magazines, parents, coaches, and teammates [39]. In a survey involving 45 male NCAA Division I college football players, the top two sources of nutrition information reported by the players were SCCs and teammates &/or friends & family [40]. In a cross-sectional study involving 200 Malaysian university students, the results showed that the main sources of nutrition information for athletes were the internet (82%), newspapers or magazines (70%), families or friends or neighbors (65%), television (60%) and coaches (52%) [41]. These readily accessible nutrition information sources, however, are potentially unreliable.

The NK of coaches, ACs, ATs and SCCs, for instance, is often insufficient for proper guidance and may lead to the dissemination of misinformation regarding sports nutrition among athletes. A recent comprehensive review of NK of coaches indicated that key sport nutrition concepts are poorly understood by most coaches [42]. A study done to assess sports NK of coaches, found coaches to have a total mean NK score of 68.4% with less than 30% of the coaches providing correct answers to some general nutrition questions regarding carbohydrates and lipids [43], .In a survey done on five varsity coaches at a Canadian college, researchers found coaches to have low NK (Mean NK with high degree of certainty: 49.5–68.3%), but still made nutrition recommendations to their athletes [44]. In another study involving 303 coaches of women’s sports at all levels of collegiate competition, researchers found that a substantial proportion of coaches engaged in some form of weight management behaviors with their athletes (for e.g., assess body composition (44.4%), encourage to lose weight by restricting intake (29.9%)), despite the fact that about 50% of the athletes have battled with issues associated with eating and weight and that only about one-third of the coaches received formal training in nutrition [45] . In addition, in a survey involving coaches (*n* = 131); ATs (*n* = 192), SCCs (*n* = 71) and athletes (*n* = 185) at NCAA Division I, II, and III institutions, adequate knowledge was found in only 35.9% of coaches (NK score ≥ 75%) [38] . In another study done on 163 UK coaching certificate level 2 and 3 hockey and netball qualified coaches, the mean total NK score for all coaches was found to be low (35.4% ± 14.8%), with no significant differences found between those who gave and did not give nutritional advice to athletes. In addition, only about one third of the coaches who provided advice received formal nutrition training [46].

Basketball is the most popular sport in Lebanon. Professional basketball players have specific dietary requirements to meet the nutrient needs of regular intensive training and ensure optimal wellbeing [47]. Optimal nutrition not only maintains the athlete’s wellbeing but also fuels the athlete’s physical performance, recovery from exercise and exercise-induced injury [1]. Research, however, suggests that dietary intake of athletes do not often meet sport nutrition recommendations due to inadequate NK. In Lebanon and in contrast to many other countries, D1B players do not get any nutrition education as part of their training schedule or school curriculum and there is no established position for sports nutritionists in basketball clubs in Lebanon. Therefore, basketball players are likely to get their nutrition information from a variety of easily available, yet potentially unreliable, sources. The aims of the study are to: 1) assess the prevalence of inadequate NK; 2) recognize the gaps in NK 3) pinpoint the main sources of nutrition information 4) understand perceptions on sports nutrition and the impact of dietitian/nutritionist on diet and performance and 4) identify the independent predictors of inadequate NK, among division I basketball players in Lebanon.

To our knowledge, the present study is the first to evaluate the NK status of D1B athletes/coaches in Lebanon. By assessing the prevalence of inadequate NK and identifying the gaps in NK and the main sources of nutrition information in this group of athletes, the need to take action by team managers, by budgeting for hiring sports nutritionists or inviting sports nutritionists to the sports club to educate D1B athletes/coaches and fill in these gaps, and school managers, by integrating nutrition into the school curriculum, may become evident. This will, in turn, help enhance the athletes’ physical health and performance.

## Methods

### Study design and recruitment methods

The study is a cross-sectional study that was conducted on professional basketball players/coaches enrolled in Division I Lebanese Championship. Subjects were recruited by targeting all basketball teams in the Men’s and the Women’s Division 1 Leagues in Lebanon. The Men’s Division 1 League is made up of ten teams consisting of 13 players each (*n* = 130) and the Women’s Division 1 League is made up of six teams consisting of nine players each (*n* = 54). All DIB players (*n* = 184 males and females) and coaches (*n* = 16 males) of the basketball teams in Lebanon were invited to participate in the study and a total of 178 out of the 184 players and 11 out of the 16 coaches participated in this study, resulting in survey response rates of 97 and 69%, respectively. To ensure representativeness of the samples of the target populations, we compared characteristics of volunteers with non-volunteers (educational background, years of professional basketball playing, …) and found responders not to differ systematically from non- responders. After obtaining the ethical approval of the Notre Dame University Institutional Review Board (NDU-IRB; Ref#: IRBF17_5_FNHS) and the different team managers, researchers visited the clubs during the competitive season (December 2017–February 2017), as per date and time set by the athlete training schedule, to recruit subjects and perform data collection.

### Data collection procedures and tool

During the visit to each club, researchers briefed basketball athletes/coaches about the study objectives and procedures and then obtained written consent from those who expressed willingness to participate. Those who signed the consent form (178 out of 184 players and 11 out of 16 coaches (survey response rates of 97 and 69%, respectively) were then asked to fill out a questionnaire, which has been used in previous studies [48, 49] and was developed from a combination of previously administered questionnaires [39, 46, 50, 51]. The questionnaire consisted of five sections: the first section of the questionnaire included questions on socio-demographic factors (SDF) (e.g., age, sex, nationality,..), academic major, professional experience (years of professional basketball playing) and previous nutrition education experience (NTREdX) (for e.g., “Have you ever taken any nutrition Courses?”; “Have you ever consulted with a dietitian/nutritionist concerning your diet?”; “Has a Dietitian/ nutritionist ever spoken to your team?”; “Have you ever received an education in relation to banned supplements?”,..) (Table 1). The second section focused on basic and sports NK and consisted of 44 statements. These 44 statements were categorized into the following themes (Macronutrients, Micronutrients, Hydration Sports Nutrition, Weight Management and Pre-exercise meal). Participants indicated their responses to these statements by answering “True,” “False,” or “Don’t Know”. Statements answered correctly were given a score of 1 and statements answered incorrectly, including those with the answer “Don’t Know,” were scored as 0, giving an overall score, referred to as the NK Score, of 0–44. The NK score was calculated for each participant by adding the total number of correct questions. So for example, a person who provided 23 correct answers will get a score of 1 on each of these responses and a score of 0 on each of the 21 incorrect answers, resulting in a NK score of 23 (out of 44) for that person. To determine NK status (adequate vs. inadequate NK), we calculated percentage of questions answered correctly as (NK score /44)*100. A percentage of questions answered correctly of ≥60% was used as indicative of a person having a NK status of adequate [49]. In reference to the example given above, percentage of questions answered correctly for that person would be (23 /44)*100 ≈ 52% and, using the cutoff point of ≥60%, this person is said to have inadequate NK (Table 2). The third section included questions on the likelihood athletes would use specific sources to obtain current nutrition information (e.g., coach, AT, ..) (Table 3). The fourth and fifth sections measured athlete’s feelings towards four statements pertaining to sports nutrition (FSNTR) and two statements pertaining to importance of having a dietitian /nutritionist on the team’s staff for enhancement of the athlete’s physical health and performance, respectively (Table 4). The third, fourth/fifth sections consisted of a five-point Likert scale ranging from 1, “Very Likely” to 5, “Very Unlikely”/ 1″Strongly Agree” to 5, “Strongly Disagree”, respectively. The “Very likely”/ “likely” and “Very Unlikely”/ “Unlikely”, Strongly Agree”/ “Agree” and “Strongly Disagree”/ “Disagree” were combined into two categories “Likely” and “Unlikely”/ “Agree” and “Disagree”, respectively, giving a total of three categories: Likely, Neutral and Unlikely /Agree, Neutral and Disagree. The questionnaire was translated to the Arabic language using professional translation services available at NDU. Prior to performing this study, a pilot study was executed, as planned for this study, to test the research protocol and questionnaire, using a random sample of 32 DIB players and 2 coaches (~ 15% of the sample size). Feedback from the pilot study was considered and appropriate adjustments were made to the research protocol and the questionnaire prior to performing this study.

**Table 1:** Sample Characteristics and Nutrition Education Experience of Division I Basketball players and Coaches

	***Basket Ball Players***				***Coaches***
	***Mean ± SD***				***Mean ± SD***
	Or n (%)				Or n (%)
	Total	Men	Women	***p***-value^a^	T***o***tal
	(***n*** = 178)	(***n*** = 126)	(***n*** = 52)		(***n*** = 11)
*Socio-demographic factors*					
**Age (years)**	27.28 ± 4.42	28.17 ± 4.18	25.13 ± 4.28	0.00	35.73 ± 9.99
**Nationality**				0.02	
Lebanese	165(92.7)	121(96.0)	44(84.6)		9(81.8)
Non-Lebanese	13(7.3)	5(4.0)	8(15.4)		2(18.2)
***Academic Major***					
**Major**^b^				0.90	
FBAE	76(43.9)	54(43.9)	22(44.0)		5(55.6)
FNAS	15(8.7)	12(9.8)	3(6.0)		–
FE	23(13.3)	15(12.2)	8(16)		–
FH	36(20.8)	26(21.1)	10(20)		4(44.4)
FAAD	13(7.5)	9(7.3)	4(8)		
FLPS	2(1.2)	1(0.8)	1(2)		
FPh	3(1.7)	3(2.4)	0(0)		
FNHS	5(2.9)	3(2.4)	2(4)		
***Professional Experience***					
**Years of professional basketball playing**	6.52 ± 4.20	5.98 ± 3.59	7.81 ± 5.22	0.11	12.36 ± 8.55
***Nutrition Education Experience***					
**Any NTR courses taken**^c^				0.51	
Yes	57(32)	38(30.2)	19(36.5)		4(36.4)
No	121(68)	88(69.8)	33(63.5)		7(63.6)
**NTR courses taken**^c^	1.63 ± 1.95	1.46 ± 0.80	1.95 ± 3.12	0.55	2.00 ± 1.00
**Basic NTR courses taken**^c^	1.63 ± 0.70	1.11 ± 0.52	1.39 ± 0.98	0.26	1.33 ± 0.58
**Advanced NTR courses** taken^c^	0.45 ± 1.55	0.35 ± 0.92	0.69 ± 2.50	0.50	0.67 ± 0.58
**Basic NTR courses with sports NTR component taken** ^c^	0.43 ± 0.72	0.35 ± 0.71	0.56 ± 0.73	0.22	1.00 ± 0.00
**Advanced NTR courses with sports NTR component taken**^c^	0.23 ± 0.75	0.30 ± 0.88	0.06 ± 0.25	0.24	0.50 ± 0.71
**Ever consulted with dietician/nutritionist?**				0.00	
Yes	54(30.3)	25(19.8)	29(55.8)		4(36.4)
No	124(69.7)	101(80.2)	23(44.2)		7(63.6)
**Ever received education on banned supplement?**				0.02	
Yes	52(29.2)	30(23.8)	22(42.3)		6(54.5)
No	126(70.8)	96(76.2)	30(57.7)		5(45.5)
**Dietician/nutritionist ever spoken to team?**				0.00	
Yes	36(20.2)	15(11.9)	21(40.4)		4(36.4)
No	142(79.8)	111(88.1)	31(59.6)		7(63.6)
**If club has a dietician/nutritionist**				0.00	
Yes	8(4.5)	6(4.8)	2(3.8)		0(0)
No	123(69.1)	77(61.1)	46(88.5)		9(81.8)
Don’t know	47(26.4)	43(34.1)	4(7.7)		2(18.2)

^a^ Refers to differences between the two sex groups; *P*-value: < 0.05 = statistical significance

^b^ *FBAE* Faculty of Business Administration and Economics, *FNAS* Faculty of Natural Applied Sciences, *FE* Faculty of Engineering, *FH* Faculty of Humanities, *FAAD* Faculty of Architecture Arts and Design, FLPS Faculty of Law and Political Science, *FPh* Faculty of Pharmacy, *FNHS* Faculty of Nursing and Health Sciences

^c^ *NTR:* Nutrition

**Table 2:** Nutrition Knowledge (NK) of DIB Players and Coaches^a^

	Basket Ball Players Mean ± SD Or n (%)				Coaches Mean ± SD Or n (%)
Basic and Sports NK Statement	Total (***n***=178)	Men (***n***-126)	Women (***n***=52)	***p***-value^b^	Total (***n***=11)
*Macronutrients* ^c^					
**Carbohydrates ( bread, potatoes..) are not as easily and rapidly digested as protein**				0.34	
Incorrect	64(36)	42(33.3)	22(42.3)		5(45.5)
Correct	114(64)	84(66.7)	30(57.7)		6(54.5)
**Carbohydrates ( bread, potatoes..) are not as easily and rapidly digested as fat**				0.06	
Incorrect	83(46.6)	65(51.6)	18(34.6)		4(36.4)
Correct	95(53.4)	61(48.4)	34(65.4)		7(63.6)
**Fiber in the diet may help to decrease constipation**				0.00	
Incorrect	86(48.3)	71(56.3)	15(28.8)		5(45.5)
Correct	92(51.7)	55(43.7)	37(71.2)		6(54.5)
**Fiber in the diet may help decrease blood cholesterol**				0.71	
Incorrect	80(44.9)	55(43.7)	25(48.1)		6(54.5)
Correct	98(55.1)	71(56.3)	27(51.9)		5(45.5)
**Fiber in the diet may help prevent cancers**				1.00	
Incorrect	75(42.1)	53(42.1)	22(42.3)		3(27.3)
Correct	103(57.9)	73(57.9)	30(57.7)		8(72.7)
**Eggs are examples of protein sources other than meats**				0.00	
Incorrect	26(14.6)	25(19.8)	1(1.9)		1(9.1)
Correct	152(85.4)	101(80.2)	51(98.1)		10(90.9)
**Legumes (beans, lentils..) are examples of protein sources other than meats**				0.01	
Incorrect	53(29.8)	30(23.8)	23(44.2)		
Correct	125(70.2)	96(76.2)	29(55.8)		11(100.0)
**Protein is the primary source of muscular energy for the athlete**				0.23	
Incorrect	145(81.5)	106(84.1)	39(75.0)		8(72.7)
Correct	33(18.5)	20(15.9)	13(25.0)		3(27.3)
**Protein is not stored in the body; therefore, it needs to be consumed everyday**				0.00	
Incorrect	60(33.7)	27(21.4)	33(63.5)		6(54.5)
Correct	118(66.3)	99(78.6)	19(36.5)		5(45.5)
**Increasing protein in the diet is necessary in order to increase muscle mass of the body**				0.01	
Incorrect	148(83.1)	98(77.8)	50(96.2)		10(90.9)
Correct	30(16.9)	28(22.2)	2(3.8)		1(9.1)
**Cheese is a good source of iron in the diet**				0.05	
Incorrect	107(60.1)	82(65.1)	25(48.1)		6(54.5)
Correct	71(39.9)	44(34.9)	27(51.9)		5(45.5)
**No more than 15% of calories in the diet should be provided by fat**				0.14	
Incorrect	142(80.2)	97(77.0)	45(88.2)		9(81.8)
Correct	35(19.8)	29(23.0)	6(11.8)		2(18.2)
*Micronutrients*					
**Iron-deficiency anemia (lack of iron) results in a decrease in the amount of oxygen that can be carried in the blood and therefore impairs athletic performance**				0.16	
Incorrect	63(35.4)	40(31.7)	23(44.2)		3(27.3)
Correct	115(64.6)	86(68.3)	29(55.8)		8(72.7)
**Vitamin supplementation is recommended for all physically active persons**				0.35	
Incorrect	141(79.2)	97(77.0)	44(84.6)		8(72.7)
Correct	37(20.8)	29(23.0)	8(15.4)		3(27.3)
**Excess vitamin supplementation may harm the physically active person**				0.00	
Incorrect	101(56.7)	84(66.7)	17(32.7)		5(45.5)
Correct	77(43.3)	42(33.3)	35(67.3)		6(54.5)
**Vitamins are a good source of energy**				0.64	
Incorrect	138(77.5)	96(76.2)	42(80.8)		6(54.5)
Correct	40(22.5)	30(23.8)	10(19.2)		5(45.5)
**Carrots are a good source of vitamin A**				0.72	
Incorrect	70(39.3)	48(38.1)	22(42.3)		3(27.3)
Correct	108(60.7)	78(61.9)	30(57.7)		8(72.7)
**Whole fat milk is a better source of vitamin D than fat free milk**				0.01	
Incorrect	120(67.4)	77(61.1)	43(82.7)		10(90.9)
Correct	59(32.6)	49(38.9)	9(17.3)		1(9.1)
**Whole fat milk is better source of vitamin D than low fat milk**				0.00	
Incorrect	116(65.2)	73(57.9)	43(82.7)		8(72.7)
Correct	62(34.8)	53(42.1)	9(17.3)		3(27.3)
**A lack of iron in the diet can result in fatigue**				0.00	
Incorrect	65(36.5)	60(47.6)	5(9.6)		2(18.2)
Correct	113(63.5)	66(52.4)	47(90.4)		9(81.8)
**A lack of iron in the diet can result in injury**				0.00	
Incorrect	81(46.3)	67(54.5)	14(26.9)		1(9.1)
Correct	94(53.7)	56(45.5)	38(73.1)		10(90.9)
*Hydration*					
**Dehydration (when you lose more fluid than you take in your body) can impair physical performance**				0.06	
Incorrect	38(21.3)	32(25.4)	6(11.5)		1(9.1)
Correct	140(78.7)	94(74.6)	46(88.5)		10(90.9)
**During activity, feeling thirsty is an adequate indicator of the need for liquid (water, sport drink...)**				0.01	
Incorrect	159(89.3)	107(84.9)	52(100.0)		11(100.0)
Correct	19(10.7)	19(15.1)	0(0.0)		
**During exercise, mass ingestion of large amounts of any fluid (water, sport drink...) is preferred over frequent ingestion of small amounts**				0.61	
Incorrect	96(53.9)	70(55.6)	26(50.0)		8(72.7)
Correct	82(46.1)	56(44.4)	26(50.0)		3(27.3)
**An athlete should drink no water during practice, but rather rinse out his or her mouth**				1.00	
Incorrect	41(23.0)	29(23.0)	12(23.1)		4(36.4)
Correct	137(77.0)	97(77.0)	40(76.9)		7(63.6)
**An athlete should drink no water during practice, but rather suck on ice cubes**				0.14	
Incorrect	46(25.8)	37(29.4)	9(17.3)		3(27.3)
Correct	132(74.2)	89(70.6)	43(82.7)		8(72.7)
**Sports drinks are the best way to replace body fluids lost during exercise**					
Incorrect	127(71.3)	90(71.4)	37(71.2)		6(54.5)
Correct	51(28.7)	36(28.6)	15(28.8)		5(45.5)
**Caffeine can increase the risk of dehydration**				0.96	
Incorrect	57(32.0)	41(32.5)	16(30.8)		4(36.4)
Correct	121(68.0)	85(67.5)	36(69.2)		7(63.6)
**An athlete involved in endurance events (eg, long distance running) should follow a considerably different diet than one participating in events of short duration (eg, sprinting)**				0.10	
Incorrect	138(77.5)	93(73.8)	45(86.5)		6(54.5)
Correct	40(22.5)	33(26.2)	7(13.5)		5(45.5)
**A physically fit person eating a nutritionally adequate diet can improve his or her performance by consuming greater amounts of nutrients**				0.00	
Incorrect	133(74.7)	85(67.5)	48(92.3)		10(90.9)
Correct	45(25.3)	41(32.5)	4(7.7)		1(9.1)
**A sound nutritional practice for athletes is to eat a wide variety of different food types everyday**				0.24	
Incorrect	72(40.4)	55(43.7)	17(32.7)		1(9.1)
Correct	106(59.6)	71(56.3)	35(67.3)		10(90.9)
**Nutrition is more important during the competitive season than during the off-season for the athlete**				0.91	
Incorrect	85(47.8)	61(48.4)	24(46.2)		2(18.2)
Correct	93(52.2)	65(51.6)	28(53.8)		9(81.8)
**What the athlete eats is only important if the athlete is trying to gain weight**				0.03	
Incorrect	45(25.3)	38(30.2)	7(13.5)		2(18.2)
Correct	133(74.7)	88(69.8)	45(86.5)		9(81.8)
**What the athlete eats is only important if the athlete is trying to lose weight**				0.68	
Incorrect	43(24.2)	32(25.4)	11(21.2)		2(18.2)
Correct	135(75.8)	94(74.6)	41(78.8)		9(81.8)
**Skipping meals is justifiable if you need to lose weight quickly**				0.00	
Incorrect	77(43.3)	64(50.8)	13(25.0)		2(18.2)
Correct	101(56.7)	62(49.2)	39(75.0)		9(81.8)
**When trying to lose weight, acidic foods such as grapefruit are of special value because they burn fat**				1.00	
Incorrect	80(44.9)	57(45.2)	23(44.2)		7(63.6)
Correct	98(55.1)	69(54.8)	29(55.8)		4(36.4)
**If trying to lose weight, carbohydrates should come from fruits and vegetables rather than from breads and pastas**				0.61	
Incorrect	65(36.5)	48(38.1)	17(32.7)		5(45.5)
Correct	113(63.5)	78(61.9)	35(67.3)		6(54.5)
**Alcohol has more calories per gram than protein**				0.24	
Incorrect	57(32.0)	37(29.4)	20(38.5)		4(36.4)
Correct	121(68.0)	89(70.6)	32(61.5)		7(63.6)
**A 90kg (200-pound) person uses about twice as many calories to run a 1.6km (1mile) as a 45kg (100-pound) person**				1.00	
Incorrect	122(68.5)	86(68.3)	36(69.2)		7(63.6)
Correct	56(31.5)	40(31.7)	16(30.8)		4(36.4)
**A person with a higher percentage of body fat may weigh less than a person of the same size with a greater muscle mass**				0.00	
Incorrect	88(49.4)	72(57.1)	16(30.8)		2(18.2)
Correct	90(50.6)	54(42.9)	36(69.2)		9(81.8)
**A high fat meal, which is slowly digested, should be avoided before athletic events**				0.19	
Incorrect	59(33.1)	46(36.5)	13(25.0)		1(9.1)
Correct	119(66.9)	80(63.5)	39(75.0)		10(90.9)
**The pre-event meal should be eaten about 3-4 hours before competition**				0.00	
Incorrect	52(29.2)	46(36.5)	6(11.5)		5(45.5)
Correct	126(70.8)	80(63.5)	46(88.5)		6(54.5)
**Foods such as potatoes are best eaten after exercise**				0.03	
Incorrect	125(70.2)	82(65.1)	43(82.7)		7(63.6)
Correct	53(29.8)	44(34.9)	9(17.3)		4(36.4)
**Foods such as honey are best eaten after exercise**				0.04	
Incorrect	115(64.6)	75(59.5)	40(76.9)		9(81.8)
Correct	63(35.4)	51(40.5)	12(23.1)		2(18.2)
**NK Score**^d^	22.08±5.32	21.89±4.94	22.55±6.16	0.18	24.18±5.25
**% NK questions answered correctly**^e^	50.18±12.08	49.74±11.22	51.25 ±14.00	0.18	54.96±11.93
**NK Status**^f^				0.03	
Adequate	35(20.1)	19(15.4)	16(31.4)		5(45.5)
Inadequate	139(79.9)	104(84.6)	35(68.6)		6(54.5)

^a^*D1B:* Division 1 Basketball

^b^ refers to differences between the two sex groups : *p*-value <0.05=statistical significance

^c^ Macronutrients (Except for water)

^d^ Equal to sum of correct answers

^e^ Equal to (NK score /44) *100

^f^ Adequate defined by ≥60% of NK questions answered correctly

**Table 3:** Likelihood to Use Resources to Obtain Current Nutrition Information among D1B players and Coaches^a^

	Basketball Players										Coaches			
Resource	Total n (%) (***n***=178)			Men n (%) (***N***=126)			Women n (%) (***N***=52)			***p*** value^b^	Total n (%) (***n***=11)			***P*** value^c^
Criteria	Likely	Neutral	Unlikely	Likely	Neutral	Unlikely	Likely	Neutral	Unlikely		Likely	Neutral	Unlikely	
Coach	64(37.2)	73(42.4)	35(20.3)	45(37.5)	58(48.3)	17(14.2)	19(36.5)	15(28.8)	18(34.6)	0.01	4(36.4)	6(54.5)	1(9.1)	0.67
Assistant Coach	69(38.8)	66(37.1)	43(24.2)	51(40.5)	55(43.7)	20(15.9)	18(34.6)	11(21.2)	23(44.2)	0.00	5(45.5)	5(45.5)	1(9.1)	0.62
Strength & Conditioning Coach	118(66.7)	45(25.4)	14(7.9)	74(59.2)	40(32.0)	11(8.8)	44(84.6)	5(9.6)	3(5.8)	0.00	10(90.9)	1(9.1)	-	0.37
Academic Journals	61(34.5)	76(42.9)	40(22.6)	38(30.4)	61(48.8)	26(20.8)	23(44.2)	15(28.8)	14(26.9)	0.05	5(45.5)	3(27.3)	3(27.3)	0.67
Magazines	48(27.1)	77(43.5)	52(29.4)	24(19.0)	68(54.0)	34(27.0)	24(47.1)	9(17.6)	18(35.3)	0.00	5(45.5)	4(36.4)	2(18.2)	0.45
Friends	47(26.4)	79(44.4)	52(29.2)	24(19.0)	65(51.6)	37(29.4)	23(44.2)	14(26.9)	15(28.8)	0.00	1(9.1)	7(63.6)	3(27.3)	0.41
College Nutrition/Health Courses	117(65.7)	45(25.3)	16(9.0)	78(61.9)	37(29.4)	11(8.7)	39(75.0)	8(15.4)	5(9.6)	0.13	9(81.8)	2(18.2)	-	0.69
Parents	66(37.3)	76(42.9)	35(19.8)	38(30.4)	61(48.8)	26(20.8)	28(53.8)	15(28.8)	9(17.3)	0.01	3(27.3)	7(63.6)	1(9.1)	0.51
Dietitian/Nutritionist	160(89.9)	13(7.3)	5(2.8)	113(89.7)	11(8.7)	2(1.6)	47(90.4)	2(3.8)	3(5.8)	0.18	10(90.9)	1(9.1)	-	0.70
Physicians	102(57.6)	55(31.1)	20(11.3)	69(54.8)	45(35.7)	12(9.5)	33(64.7)	10(19.6)	8(15.7)	0.09	6(54.5)	4(36.4)	1(9.1)	0.90
Internet	83(47.2)	71(40.3)	22(12.5)	46(36.8)	64(51.2)	15(12.0)	37(72.5)	7(13.7)	7(13.7)	0.00	7(63.6)	3(27.3)	1(9.1)	0.75
Other	-	-	1(100)	-	-	-	-	-	1(100)	-	-	-	-	-

^a^*D1B:* Division 1 basketball

^b^refers to differences between the two sex groups; *P*-value: < 0.05 = statistical significance

^c^ refers to differences between the players and coaches; *P*-value: < 0.05 = statistical significance

**Table 4:** FSNTR and impact of Dietitian/Nutritionist on Diet and Performance among D1B players and Coaches^a^

	Basket Ball Players										Coaches			
Resource	Total Sample n (%) (***n***=178)			Men n (%) (***n***=126)			Women n (%) (***N***=52)			***p*** value^b^	Total n (%) (***n***=11)			***p*** value^c^
Statement	Agree	Neutral	Disagree	Agree	Neutral	Disagree	Agree	Neutral	Disagree		Agree	Neutral	Disagree	
Caffeine has been shown to improve endurance performance (the ability/strength to continue or last)	74(41.6)	83(46.6)	21(11.8)	50(39.7)	66(52.4)	10(7.9)	24(46.2)	17(32.7)	11(21.2)	0.01	5(45.5)	5(45.5)	1(9.1)	1.00
The type of food an athlete eats affects his or her performance	154(86.5)	22(12.4)	2(1.1)	106(84.1)	19(15.1)	1(0.8)	48(92.3)	3(5.8)	1(1.9)	0.16	10(90.9)	1(9.1)	-	1.00
Learning about nutrition is not important for athletes because they eat so much food that they always get the nutrients their bodies need	91(51.1)	24(13.5)	63(35.4)	86(68.3)	21(16.7)	19(15.1)	5(9.6)	3(5.8)	44(84.6)	0.00	2(18.2)	1(9.1)	8(72.7)	0.03
Nutritional counseling would be important only to the athlete who is trying to change his or her weight	49(27.5)	54(30.3)	75(42.1)	42(33.3)	49(38.9)	35(27.8)	7(13.5)	5(9.6)	40(76.9)	0.00	1(9.1)	3(27.3)	7(63.6)	0.33
Having a dietitian/nutritionist on my team’s staff does or would help me achieve a healthy diet	121(68.0)	53(29.8)	4(2.2)	75(59.5)	49(38.9)	2(1.6)	46(88.5)	4(7.7)	2(3.8)	0.00	10(90.9)	1(9.1)	-	0.36
Having a dietitian/nutritionist on my team’s staff does or would help me improve my athletic performance	117(65.7)	54(30.3)	7(3.9)	75(59.5)	48(38.1)	3(2.4)	42(80.8)	6(11.5)	4(7.7)	0.00	9(81.8)	2(18.2)	-	0.68

^a^ *FSNTR* Feelings towards Sports Nutrition Statements and impact of Dietitian/Nutritionist on Diet and Performance; *DIB* Division 1 basketball

^b^ refers to differences between the two sex groups; *P*-value: < 0.05 = statistical significance

^c^ refers to differences between the players and coaches; *P*-value: < 0.05 = statistical significance

### Statistical analyses and power calculation

Single continuous and categorical variables were summarized as mean ± standard deviation and n (%), respectively. The T-test/ Mann-Whitney-U-test was used to determine whether the means of the two sex groups are statistically different from each other. The chi square test /Fisher’s exact test was used to describe the relationship between two categorical variables. Multiple logistic regression was used to explore the independent effects of variables representing SDF, NTREdX and FSNTR on NK status in D1B athletes. Independent variables (IVs) that were found to be associated with NK status (dependent variable) at *p <* 0.05 in the bivariate analyses were entered in blocks into the model. Model 1 examined the effect of SDF; Model 2 examined the effect of variables representing NTREdX after adjusting for the effect of SDF. Model 3 was our final model in which the effect of variables representing FSNTR was examined after controlling for the effects of variables representing SDF and NTREdX. To check whether the sample size for the study provides adequate statistical power for the study design, we calculated the power associated with the sample size for the study, using the Epi info software [52], and it was found to be 89% (unexposed: Exposed ratio = 0.48; % outcome in the unexposed group =64.4%; % outcome in the exposed group =84.9%). The data analyzed during the study can be found in Additional file 1. Statistical analyses were performed using SPSS version 22 for Windows. A *p*-value of less than 0.05 was considered to be indicative of statistical significance.

## Results

### Sample characteristics and nutrition education experience (NTREdX) of study participants

The sample consisted of 178 D1B players (~ 71% men and 29% women, mean age = 27.28 ± 4.42 years) and 11 coaches (100% men, mean age = 35.73 ± 9.99 years). The majority of basketball players and coaches were Lebanese, majored in Business and Humanities, did not take nutrition courses, have never consulted a dietitian /nutritionist concerning their diet or received an education in relation to banned supplements, reported that a dietitian/nutritionist has never spoken to their team and their club does not have a dietitian /nutritionist. Among basketball players, men were older than women with a higher proportion of men being Lebanese. A significantly lower percentage of men reported to have consulted with a dietitian /nutritionist concerning their diet, had received an education in relation to banned supplements and had a dietitian/nutritionist spoken to their team (Table 1).

### Nutrition knowledge of the study participants

The mean NK score was found to be greater for DIB coaches as compared to players (24.18 vs. 22.08, equivalent to answering slightly more than half (54.96%) vs. only about half (50.18%) of the NK questions correctly, respectively), however the difference was found to be not statistically significant. In addition, a higher percentage of the DIB coaches was found to have adequate NK than players (46% vs. ~ 20%, respectively, *p* = 0.058). Among basketball players, there was no significant difference in mean NK score between males and females however, significant differences in NK pertaining to specific nutritional statements were observed between the two sex groups. A higher proportion of males provided a correct answer to NK questions related to post-exercise meal, hydration, proteins and vitamins than females. For e.g., Foods such as potatoes and honey are best eaten after exercise; during activity, feeling thirsty is an adequate indicator of the need for liquid. On the other hand, a higher proportion of females provided a correct answer to NK questions related to pre-exercise meal, weight management, carbohydrates, and minerals than males. For e.g., the pre-event meal should be eaten about 3–4 h before competition. In regards to NK status, a significantly higher proportion of females was found to have adequate NK than males (31.4% vs. 15.4%, respectively, *p* = 0.03) (Table 2).

### Likelihood to use resources to obtain current nutrition information

Among basketball players and coaches, the top three resources that participants indicated to use to obtain current nutrition information were dietitian/nutritionist, strength and conditioning coach and college nutrition/health courses. Among basketball players, sex differences in likelihood to use resources to obtain current nutrition information were observed. Specifically, a higher proportion of women were unlikely to use a coach (W: 34.6% vs. M:14.2%, *p* = 0.01) and an assistant coach (W: 44.2% vs. M: 15.9%, *p* = 0.00) but, likely to use a strength & conditioning coach (W: 84.6% vs. M:59.2%, *p* = 0.00), magazines(W: 47.1% vs. M:19.0%, *p* = 0.00), friends(W: 44.2% vs. M:19.0%, *p* = 0.00), parents (W: 53.8% vs. M:30.4%, *p* = 0.01) and internet (W: 72.5% vs. M:36.8%, *p* = 0.00) as resources to obtain current nutrition information (Table 3).

### Feelings towards sports nutrition statements and impact of dietitian/nutritionist on diet and performance (FSNTR)

In the total sample, significant differences in perceptions on sports nutrition and impact of dietitian/nutritionist on diet and performance were observed between players and coaches. A significantly higher percentage of players agreed that learning about nutrition is not important for athletes because they eat so much food that they always get the nutrients their bodies need (Players: 51.1% vs. Coaches: 18.2%, *p* = 0.03). Among basketball players, significant sex differences were observed. A significantly higher proportion of females than males disagreed that caffeine has been shown to improve endurance performance (W: 21.2% vs. M:7.9%, *p* = 0.01), learning about nutrition is not important for athletes because they eat so much food that they always get the nutrients their bodies need (W: 84.6% vs. M: 15.1%, *p* = 0.00), nutritional counseling would be important only to the athlete who is trying to change his or her weight (W: 76.9% vs. M: 27.8%, *p* = 0.00), having a dietitian/nutritionist on the team’s staff does or would help the athlete achieve a healthy diet (W: 3.8% vs. M:1.6%, *p* = 0.00) or improve his/her athletic performance (W: 7.7% vs. M:2.4%, *p* = 0.00) (Table 4).

### Associations between NK status and socio-demographic characteristics, nutrition education experience, participants’ preferred sources of nutrition information, and perceptions on sports nutrition statements and impact of dietitian/nutritionist on diet and performance

Multiple logistic regression analysis was used to assess how well a set of variables representing SDF, NTREdX and FSNTR is able to predict NK status among D1B players and the relative contribution of each of the independent variables (SDF, NTREdX and FSNTR) in explaining the dependent variable (NK status). In Model 1, in which SDF (age, sex) were entered, males were found to be about 2.4 times more likely to have inadequate NK than females after controlling for age, with age and sex explaining about 3–5% of the variance in inadequate NK. When variables representing NTREdX (have you ever: “taken nutrition courses”, “received education on banned supplements”, “had a consultation with a dietitian/nutritionist”, “had a dietitian/ nutritionist spoken to your team”) were added to the SDF entered in Model 1 (Model 2), the observed association in Model 1 vanished and those who did not take nutrition courses / receive education on banned supplements were found to be about 2.4/3.9 times more likely to have inadequate NK than those who did, respectively, and the variance in inadequate NK explained by all the IVs in model 2 increased to about 15–23%. After additional entry of the IVs that pertain to FSNTR (“Learning about nutrition is not important for athletes because they eat so much food that they always get the nutrients their bodies need “and “Nutritional counseling would be important only to the athlete who is trying to change his or her weight”) to Model 2 (Model 3), the observed association in Model 2 between lack of nutrition courses taken and inadequate NK disappeared however, that observed between lack of education on banned supplements and inadequate NK persisted (OR≈ 3.4, *p* = 0.008) and the variance in inadequate NK explained by the all the IVs in model 3 increased only by 1–3% (Table 5).

**Table 5:** Association between NK Status and SDF, NTREdX and FSNTR, as Assessed by Multiple Logistic Regression^a^

Independent Variables	β	S.E.	***P***-value^b^	OR	95% C.I. for EXP (B)		R square
					Lower	Upper	
**Model 1**							0.033-0.053
Age	0.021	0.047	0.67	1.021	0.932	1.119	
Sex	0.887	0.414	0.03	2.427	1.077	5.467	
**Model 2**							0.148-0.233
Age	0.043	0.054	0.43	1.044	0.939	1.160	
Gender	0.306	0.484	0.53	1.357	0.526	3.503	
Nutrition Courses Taken^c^	0.855	0.428	0.05	2.351	1.016	5.443	
Consultation with a Nutritionist	0.340	0.461	0.46	1.405	0.569	3.469	
Nutritionist spoken to team	0.544	0.511	0.29	1.723	0.633	4.690	
Education on Banned supplements	1.348	0.433	0.00	3.851	1.647	9.005	
**Model 3**							0.162-0.256
Age	0.041	0.057	0.47	1.042	0.932	1.165	
Gender	-0.284	0.634	0.65	0.753	0.217	2.605	
Nutrition Courses Taken	0.750	0.449	0.10	2.117	0.877	5.109	
Consultation with a Nutritionist	0.384	0.474	0.42	1.468	0.580	3.715	
Nutritionist spoken to team	0.493	0.524	0.35	1.638	0.587	4.573	
Education on Banned supplements	1.237	0.466	0.01	3.447	1.382	8.599	
Learning about nutrition is not important for athletes ^d^							
Agree	0.628	0.635	0.32	1.874	0.540	6.499	
Neutral	0.423	0.829	0.61	1.527	0.301	7.744	
Nutrition counseling is important only to change weight ^d^							
Agree	0.718	0.627	0.25	2.051	0.600	7.010	
Neutral	0.198	0.580	0.73	1.219	0.391	3.798	

^a^ Dependent Variable: NK Status; *NK* Nutrition knowledge, *SDF* Socio-demographic factors, *NTREdX* Nutrition education experience, *FSNTR* Feelings towards Sports Nutrition Statements and impact of Dietitian/Nutritionist on Diet and Performance Statements

^b^ *P*-value: < 0.05 = statistical significance

^c^ *P*-value= 0.046

^d^ Reference group: Learning about nutrition is not important for athletes/ Nutrition counseling is important for change: Disagree

## Discussion

This is the first study to assess the NK status of athletes/coaches in Lebanon. A limited level of NK was observed; only 50%/ 55% of the NK questions were answered correctly by D1B players and coaches and 20%/ 46% of D1B players and coaches were found to have adequate NK, respectively. These findings concur with those of other research studies in which researchers consistently reported athletes and coaches to have a mean NK score between 50and 60% and poor NK. In two studies done by, Rosenbloom et al., it was found that the mean NK score of NCAA division I athletes was 5.5 ± 1.7 and 5.8 ± 1.8 out of 11, equivalent to answering 50 and 53% of the NK questions correctly, respectively [53, 54]. Gualtieri, Thompson and Stedge noted in a cross-sectional study done to assess the NK status of 100 athletes enrolled in a Division II NCAA sport that an athlete answered, on average, 11 out of 21 questions correctly (~ 52%) [36]. Torres-McGehee et al., reported an average percentage NK score for athletes at NCAA Division I, II, and III universities of about 55% and that only about 9% of athletes and ~ 36% coaches have adequate knowledge [37]. Hornstrom et al. reported that female MAC softball players answered 57% of the NK questions correctly and only 35% of them had adequate knowledge [49]. Zinn et al. reported premier rugby coaches to have responded correctly to 55.6% of all NK questions [55]. Danaher & Curley found varsity coaches to have a mean NK score, with a reported high degree of certainty in one’s answer, of less than 60% for six out of seven categories that represent nutrition recommendations to their athletes (49.5% (fluid needs) to 58.1% (training diet) [56]. On the other hand, other research studies showed way lower or higher average NK scores among athletes/coaches than those found in this study. For e.g., Jessri et al. and, Bar found that the average NK score for an athlete was about 34 and 33%, respectively [38, 57] .Cockburn et al. reported a mean total NK score for UK coaching certificate level 2 and 3 hockey and netball qualified coaches of 35.4% [44]. Alaunyte et al. and Abood, et al. and, reported athletes to have an average NK score of about 70 and 73%, respectively [25, 58]. Torres-McGehee et al., reported that the average percentage knowledge score for coaches at NCAA Division I, II, and III institutions was about 66% [37]. The differences in findings could be attributed to differences in composition of the study populations (e.g., age, years of professional experience, nutrition education experience,..) in the different studies or in cutoffs used to define adequate NK. Therefore, caution should be taken when making direct comparisons.

The finding of no significant difference in mean NK score between male and female basketball athletes was supported by findings of several studies [54, 59]. This finding, however, was contradicted by findings of other studies in which researchers found female athletes to have significantly higher NK score than their male counterparts [60, 61]. In this study, though a higher proportion of males provided a correct answer to NK questions related to post-exercise meal, hydration, proteins and vitamins than females and a higher proportion of females provided a correct answer to NK questions related to pre-exercise meal, weight management, carbohydrates, minerals and dietary supplementation than males, the percentages of males and females who provided a correct response to these questions were low. For example, only ~ 15% of the males and 0% of the females were aware that during activity thirst is not an adequate indicator of the need for fluid and lower than one fourth of the males and one fifth of the females were aware that vitamin supplementation is not recommended for all physically active persons. These findings are supported by those reported by Jessri et al., in which researchers reported women to have higher NK scores in the subcategory “weight control” [38]. On the other hand, these findings contradict with those reported in studies in which researches reported women to have higher NK scores in the subcategory “fluid”^14^ and men and women to have similar NK scores in the subcategories “recovery” [38]“supplements” [36, 38] and “weight management and hydration” [36]. The finding that a higher proportion of females was found to have adequate NK than males could be partly explained by the differences found between the two sex groups in terms of NTREdX and FSNTR.

This study showed that the top resources D1B players in Lebanon use to obtain their nutrition information included reputable resources such as dietitian/nutritionist and college nutrition/health courses and potentially disreputable ones such as the strength and conditioning coach, physician and internet. These findings are similar to those reported by Holley et al. and Hornstrom et al. in which researchers stated that the top resources athletes reported to consult were dietitian/nutritionist, college nutrition/health courses, athletic trainer and physicians [50, 51], and strength and conditioning coach [48]. These results, however, are discordant with those of previous studies citing potentially unreliable sources as the primary sources of nutrition information [37–39, 41, 59, 62]. For e.g., Gualtieri, Thompson, & Stedge noted in a cross-sectional study done to assess the NK status of athletes enrolled in a NCAA Division II institution that their primary sources of nutrition information were school, online sources, parents/friends, personal experience/personal research, coaches and athletic trainers [36].

Results of bivariate analyses (data not shown) showed that male sex, lack of NTREdX (specifically, no NTR courses taken, no consultation with a nutritionist/dietitian, no nutritionist/dietitian spoken to team and no education on banned supplements) and athlete’s perceptions on sports nutrition (specifically, “learning about nutrition is not important for athletes” and “nutrition counseling is important only to change weight”) were associated with inadequate NK of D1B players in our study. Results of multivariate analyses showed that male sex, no NTR courses taken and no education on banned supplements were associated with inadequate NK of athletes. These associations were supported by those of previous studies. In a cross-sectional study done on British adult men and women with similar age distribution, the results showed that gender had significant independent effects on knowledge score, with men having poorer NK than females [63]. In another study done on community-dwelling older adults, stepwise regression results showed that being female was associated with higher NK scores [64]. In an Australian community sample, Hendrie et al. found that female sex had a significant positive independent effect on NK [65]. Similarly, in a study done on 1040 British adults the UK, Parmenter et al. found that gender was independently associated with NK score, with women having higher scores than men [24] .This observed association between male sex and lower NK status may reflect a lower interest by males in healthy eating and weight management [63]. This would be translated into lower interest in nutrition education, resulting in a higher percentage of males having misconceptions about sports nutrition. These suggested explanations for sex differences in NK status are supported by the findings of this study; specifically, in this study, a significantly higher percentage of males agreed that “learning about nutrition is not important for athletes because they eat so much food that they always get the nutrients their bodies need” (M: 82%, F: 10%) and that “nutritional counseling would be important only to the athlete who is trying to change his or her weight” (M: 55%, F: 15%). These misperceptions on sports nutrition are clearly reflected in a significantly lower percentage of males reporting to have consulted with a dietitian /nutritionist concerning their diet (M: 20%, F: 56%). As for the independent association found between lack of nutrition education and inadequate NK, several studies supported this finding. In a study done on a convenience sample of 101 Australian Athletes to evaluate the relationship between NK and the quality of dietary intake, researchers found athletes engaged in a previous dietetic consultation to have significantly higher NK (61.6% vs. 56.6%; *p* = .034) [66]. Jessri et al., Zawila et al. Barr and Weeden et al., found that higher NK scores were associated with completion of a college-level nutrition course among collegiate athletes [38, 39, 57, 59]. Jessri et al., Wallinga et al. found that athletes who received nutrition information from a dietitian/nutritionist had higher NK scores / more confidence in making certain nutrition decisions / more understanding of nutrient periodization and were less likely to eat fast food while on group trips than those who didn’t [38, 62]. This study showed a high prevalence of inadequate NK among D1B coaches in Lebanon (54%). Yet, about one third of the players reported the coach to be a likely source of nutrition information and a high proportion of them reported to use other potentially unreliable sources such as SCC (~ 67%); physicians (~ 57%); internet (48%) …, which could partly explain the high prevalence of inadequate NK found among D1B players as well (80%). This study also demonstrated that 1) adequate NK is positively associated with NTREDX 2) the majority of the athletes and coaches approved that a dietitian does or would help them attain a healthy diet (~ 68% and ~ 91%, respectively) and improve their athletic performance (~ 66% and ~ 82%, respectively). These results, together with those of previous studies showing nutrition education to have significantly improved athletes’ NK, dietary habits and athletic performance from pretest to posttest [31–34], suggest that D1B athletes/coaches in Lebanon could significantly benefit from having a dietitian/nutritionist on the team staff. The management personnel of the basketball sports clubs in Lebanon should try to secure funding to hire dietitians/nutritionists who would be of tremendous value to athletes in terms of 1) becoming a constant source of reliable sport nutrition information 2) designing nutrition education campaigns targeted mainly towards groups of people that were found in this study to be at high risk of having inadequate NK (males, individuals with no nutrition background, …). The design of these campaigns should be based on an analysis of the incorrect responses of our study participants and the sex differences in NK found in this study, Epidemiological studies can then be conducted to test for effectiveness of these nutrition education campaigns in improving the NK of DIB players / coaches in Lebanon.

This study has several strengths. It is the first study to examine the NK status of athletes and coaches in Lebanon. It targeted all male and female D1B players and coaches and had a very high survey response rate (M: 97%; W: 96%; Coaches: 68%). Surveys were not emailed but rather completed in face to face interviews, thus optimizing data quality. This study, however, has limitations. The temporal relationships between the different exposures (NTR courses taken, …) and the outcome (NK status) cannot be established because of the cross-sectional study design. Interviews were conducted between (or at the end of) training sessions; so, considering the player’s tight schedule, often accompanied by the late finishing times and exhaustion of athletes, the responses may not have been that accurate. Data on each player’s team was not collected, therefore, comparisons in NK status among the different teams were not possible. Few questions on nutrition education experience involved a long time frame so, it is possible that athletes/ coaches were unable to recall information accurately which would have resulted in information bias. Because there are no D1B female coaches in Lebanon, a major limitation of this study is the lack of female representation in the sample of coaches. In addition, another major limitation of this study is the unequal representation of sex among recruited D1B players (n_M_ = 126 and n_F_ = 52). Equal representation of sex could not be achieved because the numbers of D1B male and female players in this study are derived from D1B male and female populations for players with unequal representation of sex (Men’s Division 1 League consists of 130 players and the Women’s Division 1 League consists of 54 players). It is important to mention, however, that the sex distribution in the sample (n _D1B players_ = 178 (n_M=_ 126 & n_F_ = 52); %_M_ = 126/178 = 71% and %_F_ = 52/178 = 29%) reflects the sex distribution in the target population (n _D1B players_ = 184 (n_M=_ 130 & n_F_ = 54); %_M_ = 130/184 = 71% and %_F_ = 54/184 = 29%).

## Conclusions

Despite the established important role of diet in enhancing the physical health and performance of athletes, our sports clubs do not have dietitians and D1B players in Lebanon do not get any nutrition education as part of their school curriculum or training schedule. This has indeed been found in this study to have resulted in a widespread prevalence of inadequate NK among D1B players and coaches in Lebanon (80% vs. 54%, respectively). Findings of this study highlight the need for 1) our basketball sports clubs to seriously consider recruiting licensed dietitians or conducting custom-made nutrition education seminars/ workshops by licensed dietitians on a regular basis and 2) the school managers in Lebanon to integrate nutrition education into the school curriculum for students in general and to regularly conduct nutrition education seminars/ workshops by licensed dietitians to student athletes. In addition, findings of the study are of tremendous clinical significance to professional basketball athletes in Lebanon in terms of improving the athletes’ physical health and performance, thereby preventing sports-related injuries. Follow-up studies are needed to assess the actual dietary intake and habits of professional basketball athletes in Lebanon in order to be able to identify health risks associated with inadequate NK.

## Supplementary Information

**Additional file 1: Dataset.** (data analyzed during the study).

## Data Availability

All data generated or analysed during this study are included in this published article [and its supplementary information files] (see Additional file 1: Dataset.xls (data analyzed during the study)).

## Abbreviations

NK: Nutrition Knowledge
D1B: Division I basketball
FLB: Lebanese Basketball federation
NCAA: National Collegiate Athletic Association
MAC: Mid-American Conference
AT: Athletic Trainer
SCC: Strength and Conditioning Coaches
SDF: Socio-Demographic Factors
NTREdX: Nutrition Education Experience
FSNTR: Feelings towards Sports Nutrition Statements
IVs: Independent Variables
